# (*E*)-1,5-Dimethyl-4-[3-(4-nitro­benz­yloxy)benzyl­ideneamino]-2-phenyl-1*H*-pyrazol-3(2*H*)-one

**DOI:** 10.1107/S1600536808035046

**Published:** 2008-10-31

**Authors:** Shou-Xin Liu, Xia Tian, Xiao-Li Zhen, Zhen-Chao Li, Jian-Rong Han

**Affiliations:** a College of Chemical & Pharmaceutical Engineering, Hebei University of Science & Technology, Shijiazhuang 050018, People’s Republic of China; b College of Sciences, Hebei University of Science & Technology, Shijiazhuang 050018, People’s Republic of China

## Abstract

In the title compound, C_25_H_22_N_4_O_4_, the central benzene ring, makes dihedral angles of 74.35 (6), 17.01 (8) and 62.19 (7)°, respectively, with the nitro­benzyl ring, the pyrazolone ring and the terminal phenyl ring. Inter­molecular C—H⋯O hydrogen bonds help to consolidate the crystal packing.

## Related literature

For the potential applications of Schiff bases, see: Jones *et al.* (1979 ▶); Larson & Pecoraro (1991 ▶); Santos *et al.* (2001 ▶). For a related structure, see: Han & Zhen (2005 ▶). For bond-length data, see: Allen *et al.* (1987 ▶).
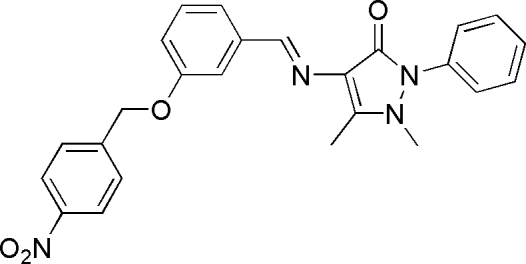


## Experimental

### 

#### Crystal data

C_25_H_22_N_4_O_4_
*M* *_r_* = 442.47
Triclinic, 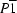

*a* = 8.003 (2) Å
*b* = 9.798 (3) Å
*c* = 14.425 (5) Å
α = 90.844 (5)°
β = 92.310 (6)°
γ = 101.202 (6)°
*V* = 1108.4 (6) Å^3^
*Z* = 2
Mo *K*α radiation
μ = 0.09 mm^−1^
*T* = 294 (2) K
0.20 × 0.18 × 0.10 mm

#### Data collection

Bruker SMART APEX CCD area-detector diffractometer
Absorption correction: multi-scan (*SADABS*; Sheldrick, 1996 ▶) *T*_min_ = 0.956, *T*_max_ = 0.991
5780 measured reflections
3898 independent reflections
2302 reflections with *I* > 2σ(*I*)
*R*_int_ = 0.026

#### Refinement

*R*[*F* ^2^ > 2σ(*F* ^2^)] = 0.046
*wR*(*F* ^2^) = 0.125
*S* = 1.01
3898 reflections
300 parameters
H-atom parameters constrained
Δρ_max_ = 0.15 e Å^−3^
Δρ_min_ = −0.16 e Å^−3^

### 

Data collection: *SMART* (Bruker, 1999 ▶); cell refinement: *SAINT* (Bruker, 1999 ▶); data reduction: *SAINT*; program(s) used to solve structure: *SHELXS97* (Sheldrick, 2008 ▶); program(s) used to refine structure: *SHELXL97* (Sheldrick, 2008 ▶); molecular graphics: *SHELXTL* (Sheldrick, 2008 ▶); software used to prepare material for publication: *SHELXTL*.

## Supplementary Material

Crystal structure: contains datablocks I, global. DOI: 10.1107/S1600536808035046/hb2829sup1.cif

Structure factors: contains datablocks I. DOI: 10.1107/S1600536808035046/hb2829Isup2.hkl

Additional supplementary materials:  crystallographic information; 3D view; checkCIF report

## Figures and Tables

**Table 1:** Hydrogen-bond geometry (Å, °)

*D*—H⋯*A*	*D*—H	H⋯*A*	*D*⋯*A*	*D*—H⋯*A*
C7—H7*B*⋯O4^i^	0.97	2.50	3.293 (3)	139

Symmetry code: (i) 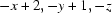.

**Fig. 1.**
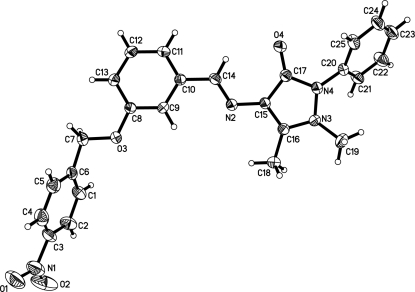
The structure of (I) with displacement ellipsoids for non-H atoms drawn at the 30% probability level.

**Fig. 2.**
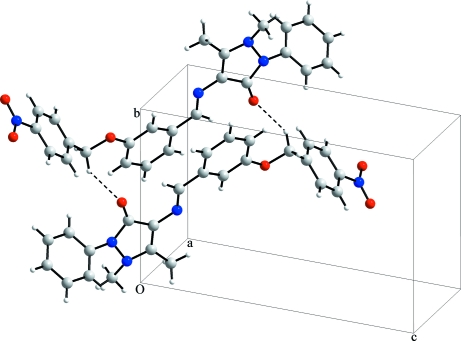
Packing diagram for (I), with H bonds drawn as dashed lines.

## supplementary crystallographic information

### Comment

There has been a steady growth of interest in the synthesis, structure, and reactivity of Schiff bases due to their potential applications in areas such as biological modelling, catalysis, and molecular magnets (Jones *et al.*, 1979; Larson & Pecoraro, 1991). One of the aims of investigating the structural chemistry of Schiff bases is to develop protein and enzyme mimics (Santos *et al.*, 2001). Among the large number of the compounds, 4-amino-1,5-dimethyl-2-phenylpyrazol-3-one forms a variety of Schiff bases with aldehydes, and the synthesis and crystal structures of them, such as (*E*)-(4)-(3-ethoxy-4-hydroxybenzylideneamino)- 1,5-dimethyl-2-phenyl-1*H*-pyrazol-3(2*H*)-one (Han & Zhen, 2005) have been reported. In present study we report the syntheses and molecular structure of the title compound, (I), (Fig. 1).

The bond lengths and angles in (I) are within their normal ranges (Allen *et al.*, 1987). The pyrazolone ring (C15—C17/N2—N4/O4) is close to planar, with an r.m.s. deviation for the fitted atoms of 0.0441 Å. It makes a dihedral angle of 49.38 (8)° with the attached phenyl ring (C20—C25). The central benzene ring (C8—C14/O3) is also planar, with an r.m.s. deviation for the fitted atoms of 0.0079 Å, and it makes dihedral angles of 74.35 (6)°, 17.01 (8)° and 62.19 (7)° with the nitrobenzyl ring (C1—C7), the pyrazolone ring (C15—C17/N2—N4/O4) and the terminal phenyl ring (C20—C25), respectively.

Packing is stabilized by weak, non-classical intermolecular C7—H7B···O4^i^ (symmetry code (i): 2 - *x*, 1 - *y*, -*z*) hydrogen bonds that form inversion related dimers, (Fig. 2, Table 1).

### Experimental

An anhydrous ethanol solution (50 ml) of 4-amino-1,5-dimethyl-2-phenylpyrazol-3-one (2.03 g, 10 mmol) was added to an anhydrous ethanol solution (100 ml) of 3-(4-nitrobenzyloxy)benzaldehyde (2.57 g, 10 mmol) and the mixture stirred at 350 K for 5 h under nitrogen, giving a yellow precipitate. The product was isolated, recrystallized from acetonitrile and then dried in a vacuum to give the pure compound in 81% yield. Yellow single crystals of (I) suitable for X-ray analysis were obtained by slow evaporation of an acetonitrile solution.

### Refinement

The H atoms were included in calculated positions and refined using a riding model approximation. Constrained C—H and N—H bond lengths and isotropic U parameters: 0.93 Å and *U*_iso_(H) = 1.2*U*_eq_(C) for C*sp*^2^—H; 0.97 Å and *U*_iso_(H) = 1.2*U*_eq_(C) for methylene C—H; 0.96 Å and *U*_iso_(H) = 1.5*U*_eq_(C) for methyl C—H.

### Crystal data

**Table:**

C_25_H_22_N_4_O_4_	*Z* = 2
*M**_r_* = 442.47	*F*(000) = 464
Triclinic, *P*1	*D*_x_ = 1.326 Mg m^−^^3^
Hall symbol: -P 1	Mo *K*α radiation, λ = 0.71073 Å
*a* = 8.003 (2) Å	Cell parameters from 2119 reflections
*b* = 9.798 (3) Å	θ = 2.4–23.9°
*c* = 14.425 (5) Å	µ = 0.09 mm^−^^1^
α = 90.844 (5)°	*T* = 294 K
β = 92.310 (6)°	Block, yellow
γ = 101.202 (6)°	0.20 × 0.18 × 0.10 mm
*V* = 1108.4 (6) Å^3^	

### Data collection

**Table:**

Bruker SMART APEX CCD area-detector diffractometer	3898 independent reflections
Radiation source: fine-focus sealed tube	2302 reflections with *I* > 2σ(*I*)
graphite	*R*_int_ = 0.026
φ and ω scans	θ_max_ = 25.0°, θ_min_ = 1.4°
Absorption correction: multi-scan (SADABS; Sheldrick, 1996)	*h* = −4→9
*T*_min_ = 0.956, *T*_max_ = 0.991	*k* = −11→11
5780 measured reflections	*l* = −16→17

### Refinement

**Table:**

Refinement on *F*^2^	Primary atom site location: structure-invariant direct methods
Least-squares matrix: full	Secondary atom site location: difference Fourier map
*R*[*F*^2^ > 2σ(*F*^2^)] = 0.046	Hydrogen site location: inferred from neighbouring sites
*wR*(*F*^2^) = 0.125	H-atom parameters constrained
*S* = 1.01	*w* = 1/[σ^2^(*F*_o_^2^) + (0.0501*P*)^2^ + 0.1798*P*] where *P* = (*F*_o_^2^ + 2*F*_c_^2^)/3
3898 reflections	(Δ/σ)_max_ < 0.001
300 parameters	Δρ_max_ = 0.15 e Å^−^^3^
0 restraints	Δρ_min_ = −0.16 e Å^−^^3^

### Special details

**Table:**

Geometry. All e.s.d.'s (except the e.s.d. in the dihedral angle between two l.s. planes) are estimated using the full covariance matrix. The cell e.s.d.'s are taken into account individually in the estimation of e.s.d.'s in distances, angles and torsion angles; correlations between e.s.d.'s in cell parameters are only used when they are defined by crystal symmetry. An approximate (isotropic) treatment of cell e.s.d.'s is used for estimating e.s.d.'s involving l.s. planes.
Refinement. Refinement of *F*^2^ against ALL reflections. The weighted *R*-factor *wR* and goodness of fit *S* are based on *F*^2^, conventional *R*-factors *R* are based on *F*, with *F* set to zero for negative *F*^2^. The threshold expression of *F*^2^ > 2σ(*F*^2^) is used only for calculating *R*-factors(gt) *etc*. and is not relevant to the choice of reflections for refinement. *R*-factors based on *F*^2^ are statistically about twice as large as those based on *F*, and *R*- factors based on ALL data will be even larger.

### Fractional atomic coordinates and isotropic or equivalent isotropic displacement parameters (Å^2^)

**Table:**

	*x*	*y*	*z*	*U*_iso_*/*U*_eq_	
N1	1.8078 (5)	0.2593 (4)	0.4869 (2)	0.0967 (10)	
N2	0.7686 (2)	0.2222 (2)	0.00080 (12)	0.0422 (5)	
N3	0.5534 (2)	−0.05908 (19)	−0.14560 (13)	0.0457 (5)	
N4	0.4987 (2)	0.05116 (19)	−0.19115 (13)	0.0456 (5)	
O1	1.9568 (4)	0.3106 (3)	0.4734 (2)	0.1346 (12)	
O2	1.7631 (4)	0.1619 (4)	0.5360 (2)	0.1486 (14)	
O3	1.2066 (2)	0.43844 (16)	0.24686 (11)	0.0527 (5)	
O4	0.5147 (2)	0.28509 (17)	−0.15735 (12)	0.0599 (5)	
C1	1.3860 (3)	0.3378 (3)	0.42021 (17)	0.0575 (7)	
H1	1.2725	0.3070	0.4338	0.069*	
C2	1.5095 (4)	0.2734 (3)	0.45952 (17)	0.0656 (8)	
H2	1.4805	0.1987	0.4986	0.079*	
C3	1.6757 (4)	0.3223 (3)	0.43939 (19)	0.0625 (8)	
C4	1.7205 (4)	0.4273 (3)	0.3794 (2)	0.0727 (9)	
H4	1.8340	0.4570	0.3654	0.087*	
C5	1.5966 (4)	0.4887 (3)	0.3400 (2)	0.0634 (8)	
H5	1.6262	0.5597	0.2981	0.076*	
C6	1.4285 (3)	0.4471 (2)	0.36121 (16)	0.0458 (6)	
C7	1.2964 (3)	0.5206 (3)	0.32183 (17)	0.0554 (7)	
H7A	1.2181	0.5341	0.3692	0.066*	
H7B	1.3500	0.6111	0.3001	0.066*	
C8	1.0789 (3)	0.4895 (2)	0.20062 (15)	0.0409 (6)	
C9	0.9887 (3)	0.4033 (2)	0.13208 (15)	0.0402 (6)	
H9	1.0185	0.3181	0.1191	0.048*	
C10	0.8547 (3)	0.4415 (2)	0.08225 (15)	0.0390 (6)	
C11	0.8139 (3)	0.5693 (2)	0.10135 (17)	0.0510 (6)	
H11	0.7245	0.5969	0.0682	0.061*	
C12	0.9050 (3)	0.6558 (3)	0.16911 (17)	0.0553 (7)	
H12	0.8768	0.7419	0.1811	0.066*	
C13	1.0377 (3)	0.6174 (2)	0.21982 (16)	0.0480 (6)	
H13	1.0982	0.6764	0.2660	0.058*	
C14	0.7563 (3)	0.3497 (2)	0.01004 (15)	0.0439 (6)	
H14	0.6827	0.3853	−0.0303	0.053*	
C15	0.6729 (3)	0.1381 (2)	−0.06885 (15)	0.0394 (6)	
C16	0.6681 (3)	−0.0010 (2)	−0.07688 (16)	0.0430 (6)	
C17	0.5600 (3)	0.1751 (3)	−0.14076 (16)	0.0441 (6)	
C18	0.7646 (3)	−0.0883 (3)	−0.02119 (19)	0.0609 (7)	
H18A	0.6885	−0.1712	−0.0035	0.091*	
H18B	0.8151	−0.0370	0.0335	0.091*	
H18C	0.8526	−0.1131	−0.0575	0.091*	
C19	0.5755 (4)	−0.1761 (3)	−0.2039 (2)	0.0815 (10)	
H19A	0.6626	−0.1452	−0.2470	0.122*	
H19B	0.4702	−0.2136	−0.2374	0.122*	
H19C	0.6085	−0.2467	−0.1657	0.122*	
C20	0.3426 (3)	0.0258 (2)	−0.24550 (15)	0.0429 (6)	
C21	0.2107 (3)	−0.0796 (3)	−0.22521 (17)	0.0546 (7)	
H21	0.2218	−0.1376	−0.1758	0.066*	
C22	0.0611 (3)	−0.0984 (3)	−0.2790 (2)	0.0678 (8)	
H22	−0.0270	−0.1724	−0.2673	0.081*	
C23	0.0401 (4)	−0.0105 (3)	−0.34928 (19)	0.0680 (8)	
H23	−0.0626	−0.0221	−0.3838	0.082*	
C24	0.1725 (4)	0.0950 (3)	−0.36802 (19)	0.0677 (8)	
H24	0.1593	0.1557	−0.4155	0.081*	
C25	0.3243 (3)	0.1123 (3)	−0.31772 (17)	0.0590 (7)	
H25	0.4148	0.1824	−0.3324	0.071*	

### Atomic displacement parameters (Å^2^)

**Table:**

	*U*^11^	*U*^22^	*U*^33^	*U*^12^	*U*^13^	*U*^23^
N1	0.086 (2)	0.139 (3)	0.079 (2)	0.064 (2)	−0.0193 (19)	−0.031 (2)
N2	0.0413 (11)	0.0414 (12)	0.0430 (12)	0.0069 (9)	−0.0026 (9)	0.0009 (9)
N3	0.0454 (12)	0.0396 (12)	0.0529 (12)	0.0122 (9)	−0.0050 (10)	−0.0050 (10)
N4	0.0458 (12)	0.0429 (12)	0.0461 (12)	0.0060 (9)	−0.0088 (9)	−0.0012 (10)
O1	0.0696 (18)	0.178 (3)	0.167 (3)	0.0610 (19)	−0.0341 (18)	−0.045 (2)
O2	0.151 (3)	0.210 (4)	0.117 (3)	0.116 (3)	−0.005 (2)	0.049 (2)
O3	0.0569 (10)	0.0463 (10)	0.0548 (11)	0.0148 (8)	−0.0200 (8)	−0.0083 (8)
O4	0.0688 (12)	0.0429 (11)	0.0664 (12)	0.0114 (9)	−0.0219 (9)	0.0046 (9)
C1	0.0452 (15)	0.0752 (19)	0.0525 (16)	0.0124 (14)	0.0027 (13)	0.0078 (14)
C2	0.073 (2)	0.090 (2)	0.0424 (16)	0.0360 (17)	0.0040 (14)	0.0140 (14)
C3	0.0520 (18)	0.094 (2)	0.0489 (17)	0.0370 (17)	−0.0127 (14)	−0.0179 (16)
C4	0.0469 (18)	0.086 (2)	0.083 (2)	0.0065 (16)	0.0052 (16)	−0.0171 (19)
C5	0.0579 (19)	0.0587 (18)	0.071 (2)	0.0040 (15)	0.0047 (15)	−0.0012 (14)
C6	0.0453 (15)	0.0501 (15)	0.0398 (14)	0.0061 (12)	−0.0059 (11)	−0.0075 (12)
C7	0.0619 (17)	0.0515 (16)	0.0502 (16)	0.0097 (13)	−0.0169 (13)	−0.0061 (12)
C8	0.0443 (14)	0.0389 (14)	0.0392 (13)	0.0080 (11)	−0.0015 (11)	0.0033 (11)
C9	0.0441 (14)	0.0319 (13)	0.0444 (14)	0.0074 (11)	0.0001 (11)	−0.0016 (11)
C10	0.0412 (14)	0.0371 (14)	0.0385 (13)	0.0066 (11)	0.0011 (11)	0.0039 (11)
C11	0.0568 (16)	0.0473 (15)	0.0524 (16)	0.0207 (13)	−0.0074 (12)	0.0027 (12)
C12	0.0704 (18)	0.0422 (15)	0.0566 (17)	0.0211 (14)	−0.0041 (14)	−0.0063 (13)
C13	0.0572 (16)	0.0411 (15)	0.0457 (15)	0.0117 (12)	−0.0049 (12)	−0.0067 (11)
C14	0.0427 (14)	0.0462 (16)	0.0429 (14)	0.0099 (11)	−0.0038 (11)	0.0055 (11)
C15	0.0392 (13)	0.0386 (14)	0.0390 (13)	0.0055 (11)	−0.0042 (11)	0.0009 (11)
C16	0.0377 (13)	0.0432 (15)	0.0481 (15)	0.0091 (11)	−0.0009 (11)	−0.0016 (11)
C17	0.0427 (14)	0.0426 (15)	0.0451 (15)	0.0041 (12)	−0.0003 (11)	0.0031 (12)
C18	0.0627 (17)	0.0536 (16)	0.0697 (19)	0.0229 (14)	−0.0131 (14)	−0.0031 (14)
C19	0.081 (2)	0.075 (2)	0.094 (2)	0.0380 (17)	−0.0233 (18)	−0.0422 (18)
C20	0.0447 (14)	0.0440 (14)	0.0387 (14)	0.0075 (12)	−0.0061 (11)	−0.0029 (11)
C21	0.0507 (16)	0.0564 (16)	0.0528 (16)	0.0015 (13)	−0.0078 (13)	0.0144 (13)
C22	0.0507 (17)	0.0715 (19)	0.075 (2)	−0.0033 (14)	−0.0114 (15)	0.0185 (16)
C23	0.0587 (18)	0.074 (2)	0.0666 (19)	0.0077 (16)	−0.0233 (14)	0.0060 (16)
C24	0.082 (2)	0.0652 (19)	0.0503 (17)	0.0049 (17)	−0.0183 (15)	0.0127 (14)
C25	0.0632 (18)	0.0593 (17)	0.0465 (16)	−0.0056 (14)	−0.0095 (13)	0.0063 (13)

### Geometric parameters (Å, °)

**Table:**

N1—O2	1.204 (4)	C9—H9	0.9300
N1—O1	1.226 (4)	C10—C11	1.380 (3)
N1—C3	1.474 (4)	C10—C14	1.464 (3)
N2—C14	1.278 (3)	C11—C12	1.372 (3)
N2—C15	1.394 (3)	C11—H11	0.9300
N3—C16	1.362 (3)	C12—C13	1.380 (3)
N3—N4	1.404 (3)	C12—H12	0.9300
N3—C19	1.454 (3)	C13—H13	0.9300
N4—C17	1.399 (3)	C14—H14	0.9300
N4—C20	1.425 (3)	C15—C16	1.360 (3)
O3—C8	1.376 (3)	C15—C17	1.444 (3)
O3—C7	1.422 (3)	C16—C18	1.483 (3)
O4—C17	1.225 (3)	C18—H18A	0.9600
C1—C6	1.377 (3)	C18—H18B	0.9600
C1—C2	1.380 (3)	C18—H18C	0.9600
C1—H1	0.9300	C19—H19A	0.9600
C2—C3	1.368 (4)	C19—H19B	0.9600
C2—H2	0.9300	C19—H19C	0.9600
C3—C4	1.358 (4)	C20—C21	1.370 (3)
C4—C5	1.366 (4)	C20—C25	1.373 (3)
C4—H4	0.9300	C21—C22	1.380 (3)
C5—C6	1.376 (3)	C21—H21	0.9300
C5—H5	0.9300	C22—C23	1.366 (4)
C6—C7	1.489 (3)	C22—H22	0.9300
C7—H7A	0.9700	C23—C24	1.367 (4)
C7—H7B	0.9700	C23—H23	0.9300
C8—C9	1.375 (3)	C24—C25	1.370 (4)
C8—C13	1.383 (3)	C24—H24	0.9300
C9—C10	1.382 (3)	C25—H25	0.9300
			
O2—N1—O1	124.4 (4)	C11—C12—H12	119.4
O2—N1—C3	118.4 (4)	C13—C12—H12	119.4
O1—N1—C3	117.2 (4)	C12—C13—C8	118.6 (2)
C14—N2—C15	120.26 (19)	C12—C13—H13	120.7
C16—N3—N4	106.75 (17)	C8—C13—H13	120.7
C16—N3—C19	123.4 (2)	N2—C14—C10	122.0 (2)
N4—N3—C19	116.3 (2)	N2—C14—H14	119.0
C17—N4—N3	109.20 (18)	C10—C14—H14	119.0
C17—N4—C20	123.36 (19)	C16—C15—N2	123.0 (2)
N3—N4—C20	119.91 (18)	C16—C15—C17	107.85 (19)
C8—O3—C7	117.38 (18)	N2—C15—C17	129.1 (2)
C6—C1—C2	121.0 (2)	C15—C16—N3	110.6 (2)
C6—C1—H1	119.5	C15—C16—C18	128.7 (2)
C2—C1—H1	119.5	N3—C16—C18	120.7 (2)
C3—C2—C1	118.2 (3)	O4—C17—N4	123.6 (2)
C3—C2—H2	120.9	O4—C17—C15	131.6 (2)
C1—C2—H2	120.9	N4—C17—C15	104.81 (19)
C4—C3—C2	122.0 (3)	C16—C18—H18A	109.5
C4—C3—N1	120.2 (3)	C16—C18—H18B	109.5
C2—C3—N1	117.8 (3)	H18A—C18—H18B	109.5
C3—C4—C5	119.1 (3)	C16—C18—H18C	109.5
C3—C4—H4	120.4	H18A—C18—H18C	109.5
C5—C4—H4	120.4	H18B—C18—H18C	109.5
C4—C5—C6	121.0 (3)	N3—C19—H19A	109.5
C4—C5—H5	119.5	N3—C19—H19B	109.5
C6—C5—H5	119.5	H19A—C19—H19B	109.5
C5—C6—C1	118.6 (2)	N3—C19—H19C	109.5
C5—C6—C7	120.3 (2)	H19A—C19—H19C	109.5
C1—C6—C7	121.0 (2)	H19B—C19—H19C	109.5
O3—C7—C6	108.51 (19)	C21—C20—C25	120.1 (2)
O3—C7—H7A	110.0	C21—C20—N4	121.3 (2)
C6—C7—H7A	110.0	C25—C20—N4	118.6 (2)
O3—C7—H7B	110.0	C20—C21—C22	119.1 (2)
C6—C7—H7B	110.0	C20—C21—H21	120.5
H7A—C7—H7B	108.4	C22—C21—H21	120.5
C9—C8—O3	115.52 (19)	C23—C22—C21	121.2 (3)
C9—C8—C13	120.2 (2)	C23—C22—H22	119.4
O3—C8—C13	124.3 (2)	C21—C22—H22	119.4
C8—C9—C10	121.0 (2)	C22—C23—C24	118.9 (3)
C8—C9—H9	119.5	C22—C23—H23	120.5
C10—C9—H9	119.5	C24—C23—H23	120.5
C11—C10—C9	118.8 (2)	C23—C24—C25	120.8 (3)
C11—C10—C14	119.8 (2)	C23—C24—H24	119.6
C9—C10—C14	121.4 (2)	C25—C24—H24	119.6
C12—C11—C10	120.2 (2)	C24—C25—C20	119.8 (2)
C12—C11—H11	119.9	C24—C25—H25	120.1
C10—C11—H11	119.9	C20—C25—H25	120.1
C11—C12—C13	121.2 (2)		
			
C16—N3—N4—C17	−9.3 (2)	C15—N2—C14—C10	−180.0 (2)
C19—N3—N4—C17	−151.5 (2)	C11—C10—C14—N2	−167.5 (2)
C16—N3—N4—C20	−160.00 (19)	C9—C10—C14—N2	12.7 (3)
C19—N3—N4—C20	57.8 (3)	C14—N2—C15—C16	−173.7 (2)
C6—C1—C2—C3	1.0 (4)	C14—N2—C15—C17	3.7 (3)
C1—C2—C3—C4	−2.9 (4)	N2—C15—C16—N3	174.59 (19)
C1—C2—C3—N1	175.8 (2)	C17—C15—C16—N3	−3.3 (3)
O2—N1—C3—C4	−176.4 (3)	N2—C15—C16—C18	−3.9 (4)
O1—N1—C3—C4	3.8 (4)	C17—C15—C16—C18	178.2 (2)
O2—N1—C3—C2	4.9 (5)	N4—N3—C16—C15	7.7 (3)
O1—N1—C3—C2	−174.9 (3)	C19—N3—C16—C15	146.6 (2)
C2—C3—C4—C5	1.9 (4)	N4—N3—C16—C18	−173.6 (2)
N1—C3—C4—C5	−176.7 (3)	C19—N3—C16—C18	−34.8 (4)
C3—C4—C5—C6	1.0 (4)	N3—N4—C17—O4	−170.3 (2)
C4—C5—C6—C1	−2.8 (4)	C20—N4—C17—O4	−20.9 (4)
C4—C5—C6—C7	176.5 (2)	N3—N4—C17—C15	7.2 (2)
C2—C1—C6—C5	1.8 (4)	C20—N4—C17—C15	156.7 (2)
C2—C1—C6—C7	−177.4 (2)	C16—C15—C17—O4	174.8 (3)
C8—O3—C7—C6	−179.61 (19)	N2—C15—C17—O4	−2.9 (4)
C5—C6—C7—O3	101.9 (3)	C16—C15—C17—N4	−2.5 (2)
C1—C6—C7—O3	−78.9 (3)	N2—C15—C17—N4	179.8 (2)
C7—O3—C8—C9	−176.4 (2)	C17—N4—C20—C21	−117.6 (3)
C7—O3—C8—C13	2.9 (3)	N3—N4—C20—C21	28.8 (3)
O3—C8—C9—C10	178.4 (2)	C17—N4—C20—C25	60.5 (3)
C13—C8—C9—C10	−0.9 (3)	N3—N4—C20—C25	−153.1 (2)
C8—C9—C10—C11	1.1 (3)	C25—C20—C21—C22	0.9 (4)
C8—C9—C10—C14	−179.1 (2)	N4—C20—C21—C22	179.0 (2)
C9—C10—C11—C12	−0.4 (4)	C20—C21—C22—C23	−2.8 (4)
C14—C10—C11—C12	179.8 (2)	C21—C22—C23—C24	2.2 (5)
C10—C11—C12—C13	−0.4 (4)	C22—C23—C24—C25	0.3 (5)
C11—C12—C13—C8	0.5 (4)	C23—C24—C25—C20	−2.2 (4)
C9—C8—C13—C12	0.2 (3)	C21—C20—C25—C24	1.6 (4)
O3—C8—C13—C12	−179.1 (2)	N4—C20—C25—C24	−176.6 (2)

### Hydrogen-bond geometry (Å, °)

**Table:**

*D*—H···*A*	*D*—H	H···*A*	*D*···*A*	*D*—H···*A*
C7—H7B···O4^i^	0.97	2.50	3.293 (3)	139

Symmetry codes: (i) −*x*+2, −*y*+1, −*z*.
